# RANK-ligand (RANKL) expression in young breast cancer patients and during pregnancy

**DOI:** 10.1186/s13058-015-0538-7

**Published:** 2015-02-21

**Authors:** Hatem A Azim, Fedro A Peccatori, Sylvain Brohée, Daniel Branstetter, Sherene Loi, Giuseppe Viale, Martine Piccart, William C Dougall, Giancarlo Pruneri, Christos Sotiriou

**Affiliations:** 1 Breast Cancer Translational Research Laboratory (BCTL) J. C. Heuson, Institut Jules Bordet, Université Libre de Bruxelles, Boulevard de Waterloo 121, Brussels, BE 1000 Belgium; 2 Department of Medicine, BrEAST Data Centre, Institut Jules Bordet, Université Libre de Bruxelles (ULB), Brussels, 1 Belgium; 3 Fertility and Procreation Unit, Department of Gynecologic Oncology, European Institute of Oncology, via Ripamonti 435, Milan, 20141 Italy; 4 Department of Pathology, Amgen Inc., 1201 Amgen Ct. West, Seattle, WA 98119 USA; 5 Translational Breast Can cer Genomic Lab, Cancer Therapeutics Program, Peter MacCallum Cancer Centre, 2 St Andrews Place, East Melbourne, VIC 3002 Australia; 6 Department of Pathology, European Institute of Oncology and University of Milan, via Ripamonti 435, Milan, 20141 Italy; 7 Department of Medical Oncology, Institut Jules Bordet, Université Libre de Bruxelles (ULB), Brussels, 1 Belgium; 8 Therapeutic Innovation Unit, Amgen Inc, 1201 Amgen Ct. West, Seattle, WA 98119 USA

## Abstract

**Introduction:**

RANKL is important in mammary gland development during pregnancy and mediates the initiation and progression of progesterone-induced breast cancer. No clinical data are available on the effect of pregnancy on RANK/RANKL expression in young breast cancer patients.

**Methods:**

We used our previously published dataset of 65 pregnant and 130 matched young breast cancer patients with full clinical, pathological, and survival information. 85% of patients had available transcriptomic data as well. RANK/RANKL expression by immunohistochemistry using H-score on the primary tumor and adjacent normal tissue was performed. We examined the difference in expression of RANK/RANKL between pregnant and non-pregnant patients and their association with clinicopathological features and prognosis. We also evaluated genes and pathways associated with RANK/RANKL expression on primary tumors.

**Results:**

RANKL but not RANK expression was more prevalent in the pregnant group, both on the tumor and adjacent normal tissue, independent of other clinicopathological factors (both *P* <0.001). 18.7% of pregnant and 5.3% of non-pregnant patients had tumors showing ≥10% of cells with 3+ RANKL expression. RANKL expression was significantly higher in progesterone receptor-positive, and luminal A-like tumors, with negative correlation with Ki-67 (all *P* <0.001). On the contrary, RANK expression was higher in triple negative tumors (*P* <0.001). Using false discovery rate <0.05, 151 and 1,207 genes were significantly correlated with tumor-expressed RANKL and RANK expression by immunohistochemistry, respectively. High RANKL expression within primary tumor was associated with pathways related to mammary gland development, bone resorption, T-cell proliferation and regulation of chemotaxis, while RANK expression was associated with immune response and proliferation pathways. At a median follow-up of 65 months, neither RANK nor RANKL expression within tumor was associated with disease free survival in pregnant or non-pregnant group.

**Conclusions:**

Pregnancy increases RANKL expression both in normal breast and primary tumors. These results could guide further development of RANKL-targeted therapy.

**Electronic supplementary material:**

The online version of this article (doi:10.1186/s13058-015-0538-7) contains supplementary material, which is available to authorized users.

## Introduction

Receptor activator for nuclear factor κB ligand (RANKL) is a key factor in bone resorption. It binds to receptor activator for nuclear factor κB (RANK) on the osteoclast to promote osteoclastogenesis, which results in bone destruction, osteoporosis and osseous metastasis [1]. Targeting the RANK/RANKL pathway emerged as a rational strategy to arrest this process, and the anti-RANKL monoclonal antibody denosumab is currently approved in managing osteoporosis and preventing skeletal-related events secondary to bone metastases [2,3].

Moreover, RANKL and its receptor have been shown to play a pivotal role in mammary gland development and in the increase of mammary stem cell pool during pregnancy [4]. Preclinical studies showed that RANKL is a major paracrine effector of progesterone’s mitogenic action in the mammary epithelium [5,6]. More recently, data in humans suggested that RANKL expression fluctuates with serum progesterone, both on normal and malignant breast tissue [7]. Increased mammary tumor formation was observed in transgenic mice with gain of function in RANK following pregnancy or in wildtype mice following treatment with progesterone, a process that was arrested using a RANKL inhibitor [6]. Furthermore, RANKL was shown to be vital in mediating distant metastasis in breast cancer mice models [6,8]. Together, this evidence points to a fundamental role of RANK/RANKL signaling in breast carcinogenesis.

Breast cancer arising at a young age is known to be biologically distinct, yet little progress has been made in identifying potential treatment targets [9]. Previous analysis by our group has suggested an association between breast cancer arising at young age and high RANKL mRNA expression [10]. On the other hand, young women are at a higher risk of breast cancer shortly after pregnancy and pregnancy-associated breast cancer is known to have poor prognosis [11,12]. While preclinical data have suggested a potential role of RANKL in mediating cancer initiation and progression associated with pregnancy [4], supporting clinical data in pregnant cancer patients are lacking.

In the current study, we evaluated for the first time the expression of RANK and RANKL using immunohistochemistry in young and pregnant breast cancer patients. Based on preclinical observations, we hypothesized that pregnancy would increase RANKL expression. We also evaluated gene expression patterns and activated pathways associated with RANK and RANKL expression.

## Methods

### Study population

A total of 195 patients with primary breast cancer were included in this analysis, of whom 65 were diagnosed during pregnancy. All patients were diagnosed and managed at the European Institute of Oncology (IEO) in Milan from 1996 to 2010. Information on the patient’s characteristics and outcome was published previously [13]. Briefly, each pregnant patient was matched to two nonpregnant breast cancer patient controls according to age, tumor size, nodal status, date of diagnosis and whether neoadjuvant therapy was administered. All patients provided their consent to use their tissue samples for research purposes as per the IEO institutional policies. The study of biological features including genomic analysis was approved by the Ethics Committee of Institut Jules Bordet (Number 1782).

### Immunohistochemical staining

Formalin-fixed tissues of primary breast surgeries were used for RANK and RANKL evaluation. For each patient, a hematoxylin and eosin-stained slide along with representative slides of the primary tumor and adjacent normal tissue (>1 cm from tumor) were shipped to Amgen Laboratories (Seattle, WA, USA) for immunohistochemical staining of RANK (N-1H8) and RANKL (M366) as described previously [14,15], blinded to clinical information.

For each epitope, the staining score for tumor cells and adjacent normal epithelial cells was recorded separately. The percentage of immunostaining and the staining intensity (0, negative; 1+, weak; 2+, moderate; and 3+, strong) were recorded. An H-score was calculated using the following formula:

$$ \begin{array}{c}\hfill \mathrm{H}\hbox{-} \mathrm{score} = \left(\%\ \mathrm{of}\ \mathrm{cells}\ \mathrm{of}\ \mathrm{weak}\ \mathrm{intensity} \times 1\right) + \left(\mathrm{percentage}\ \mathrm{of}\ \mathrm{cells}\ \mathrm{of}\ \mathrm{moderate}\ \mathrm{intensity} \times 2\right)\ \hfill \\ {}\hfill + \left(\mathrm{percentage}\ \mathrm{of}\ \mathrm{cells}\ \mathrm{of}\ \mathrm{strong}\ \mathrm{intensity} \times 3\right)\hfill \end{array} $$

The maximum H-score would be 300, corresponding to 100% of cells with strong intensity.

Evaluation of the histological subtype, histological grade and conventional breast cancer markers including estrogen receptor (ER), progesterone receptor (PgR), human epidermal growth factor receptor 2 (HER2) and Ki67 were performed at IEO. Breast cancer subtypes were defined using immunohistochemical surrogates as follows: luminal A like – ER and/or PgR(+), HER2(–), Ki67 < 20%; luminal B like – ER and/or PgR(+), HER2(–), Ki67 ≥ 20; triple negative – ER, PgR and HER2(–), irrespective of Ki67 score; and HER2 – HER2(+), irrespective of ER, PgR or Ki67.

### Gene expression profiling

Gene expression profiling using Affymetrix on the primary tumors of 85% of the included patients was published previously [16] and is publically available on Gene Expression Omnibus [GEO:GSE53031] [17].

To evaluate gene expression differences according to RANK and RANKL expression, we performed a linear regression model – testing genes that are associated with RANK, RANKL H-score; all treated as continuous variables. To control for multiple testing, we used the false discovery rate approach [18]. Genes presenting false discovery rate <0.05 were considered significantly associated with RANK or RANKL expression. We used a gene-set enrichment analysis (GSEA) to evaluate pathways associated with RANK or RANKL expression using the hypergeometric probabilities as published previously [19].

### Statistical analyses

We evaluated the difference in expression of RANK/RANKL H-score tested as a continuous variable on tumor and adjacent normal tissue between pregnant and nonpregnant patients. We also evaluated correlations between RANK/RANKL expression and the variables tumor size, nodal status, ER, PgR, HER2, Ki67 and breast cancer subtype using the Pearson chi-squared test. To evaluate factors that were independently associated with RANK/RANKL expression, a linear regression model was constructed including all of the aforementioned variables.

We evaluated the association between tumor RANK and RANKL expression as a continuous variable and disease-free survival in a Cox regression model, for pregnant, nonpregnant and all patients combined.

Analyses were performed using SAS version 9.2 (SAS Institute, Cary, NC, USA) and R version 2.12.2 [20]. All tests were two-sided.

## Results

Table 1 summarizes the patients’ characteristics. As reported previously [13], no differences in clinicopathological features or breast cancer subtypes were observed between pregnant and nonpregnant patients. The median age was 36 years (range: 26 to 48 years). Information on prior parity and menstrual status at tumor sampling was not available.

**Table 1:** **Patient characteristics and association with RANK and RANKL expression by immunohistochemistry**

	**Pregnant (** ***n*** **= 65)**	**Nonpregnant (** ***n*** **= 130)**	**Mean RANKL H**-**score on primary tumor (** ***P*** **value)** ^**a**^	**Mean RANK H**-**score on primary tumor (** ***P*** **value)**
Tumor size				
≤ 2 cm	26 (40%)	52 (40%)	28.21	10.08
> 2 cm or pTx^b^	38 (60%)	78 (60%)	8.63 (*P* = 0.01)	17.50 (*P* = 0.15)
Nodal status				
Negative	28 (43.1%)	56 (43.1%)	24.51	14.12
Positive or pNx^b^	37 (56.9%)	74 (56.9%)	9.04 (*P* = 0.03)	14.36 (*P* = 0.96)
Histological grade^c^				
I	4 (6.2%)	4 (3.1%)	70	7.5
II	21 (32.3%)	43 (33.1%)	29.92	3.82
III	36 (55.3%)	68 (52.3%)	6.25 (*P* < 0.001)	22.2 (*P* < 0.001)
ER				
Positive	43 (66.1%)	96 (73.8%)	20.43	5.43
Negative	22 (33.9%)	34 (26.2%)	5 (*P* = 0.05)	36.71 (*P* < 0.001)
PgR				
Positive	42 (64.6%)	85 (65.4%)	24.11	5.09
Negative	23 (35.4%)	45 (34.6%)	0.67 (*P* < 0.001)	31.76 (*P* < 0.001)
HER2				
Positive	11 (16.9%)	23 (17.7%)	3.18	10.82
Negative	54 (83.1%)	107 (82.3%)	18.79 (*P* = 0.1)	14.95 (*P* = 0.53)
PIK3CA mutation^d^				
Yes	10 (15.4%)	27 (20.7%)	24.03	6.3
No	52 (84.7%)	102 (79.3%)	12.47 (*P* = 0.18)	15.63 (*P* = 0.13)

ER, estrogen receptor; HER2, human epidermal growth factor receptor 2; PgR, progesterone receptor; RANK, receptor activator for nuclear factor κB; RANKL, receptor activator for nuclear factor κB ligand. ^a^Not assessable in one patient in the pregnant group. ^b^A total of six patients had pathological tumor size is not evaluable (pTx) or pathological nodal status is not evaluable (pNx) (two pregnant and four nonpregnant). ^c^Not assessable in 19 patients (four pregnant and 15 nonpregnant). ^d^Not assessable in four patients (one pregnant and three nonpregnant). pTx (pathological tumor size is not evaluable) or pNx (pathological nodal status is not evaluable).

### RANK and RANKL expression by immunohistochemistry and association with pregnancy and clinicopathological features

**Figure 1:**
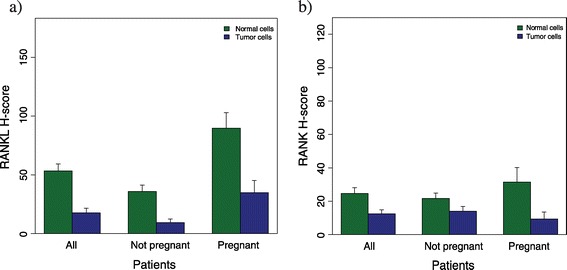
**Differences in RANK and RANKL expression between pregnant and nonpregnant breast cancer patients. (a)** Expression of RANKL by immunohistochemistry using the H-score (*y* axis) in all patients, pregnant patients and nonpregnant patients both in primary tumor (blue) and adjacent normal breast epithelial cells (green). Mean RANKL H-score was higher in normal breast epithelial cells compared with tumor cells; 50.19 versus 16.13, *P* <0.001 in all patients; 32.88 versus 8.06, *P* <0.001 in nonpregnant patients; and 87.29 versus 32.53 in pregnant patients, *P* <0.001. **(b)** Expression of RANK by immunohistochemistry using the H-score (*y* axis) in all patients, pregnant patients and nonpregnant patients both in primary tumor (blue) and adjacent normal breast epithelial cells (green). Mean RANK H-score was higher in normal breast epithelial cells compared with tumor cells; 24.65 versus 12.48, *P* = 0.003 in all patients; 21.72 versus 13.95, *P* = 0.07 in nonpregnant patients; and 31.52 versus 9.41 in pregnant patients, *P* = 0.016. RANK, receptor activator for nuclear factor κB; RANKL, receptor activator for nuclear factor κB ligand.

**Figure 2:**
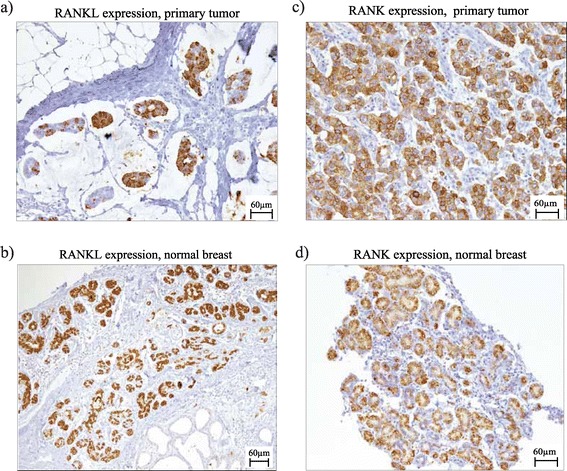
**Examples of RANK and RANKL immunohistochemical staining on primary breast tumors and normal breast tissue. (a)** RANKL expression on the primary tumor in a patient diagnosed with breast cancer during pregnancy (H-score: 270, 80% of cells showing RANKL expression score 3+). **(b)** RANKL expression on adjacent normal epithelial cells in a patient diagnosed with breast cancer during pregnancy (H-score: 265, 80% of cells showing RANKL expression score 3+). **(c)** RANK expression on the primary tumor in a young breast cancer patient not diagnosed during pregnancy (H-score: 140, 20% of cells showing RANK expression score 3+). **(d)** RANK expression on adjacent normal epithelial cells in a patient diagnosed with breast cancer during pregnancy (H-score: 210, 40% of cells showing RANK expression score 3+). RANK, receptor activator for nuclear factor κB; RANKL, receptor activator for nuclear factor κB ligand.

RANKL staining was performed on 194 primary tumors (99%) and 176 adjacent normal tissues (90%); the results were positively correlated (*r* = 0.38, *P* < 0.001). The mean RANKL expression on normal epithelial cells was higher compared with that on tumor cells (50.19 vs. 16.13, *P* <0.001) (Figure 1a). Patients diagnosed during pregnancy had significantly higher RANKL expression both on tumor cells (32.53 vs. 8.06, *P* <0.001) and adjacent normal tissue (87.29 vs. 32.88, *P* <0.001) (Figure 1a). In total, 18.7% of pregnant patients and 5.3% of nonpregnant patients had ≥10% of cells showing strong RANKL staining (score 3+) on primary tumor, while 55.3% and 29.1% of pregnant and nonpregnant patients had at least 10% of cells 3+ on adjacent normal tissue (Figure 2a,b). Additional file 1 shows examples of different staining intensities of RANKL. Among pregnant patients, a slightly lower expression of RANKL in both tumor and adjacent normal epithelial cells was observed in patients diagnosed in the third trimester of pregnancy (Figure S2a,b in Additional file 2).

RANK staining was performed on all 195 primary tumors and 181 adjacent normal tissue samples (92.8%) and both results were positively correlated (*r* = 0.27, *P* < 0.001). The mean RANK H-score was higher for normal versus tumor tissue (23.65 vs. 14.25, *P* = 0.003) (Figure 1b). No significant differences in RANK expression were observed between pregnant and nonpregnant patients; either on tumor tissue (13.74 vs. 14.51, *P* = 0.88) or adjacent normal epithelial cells (32.29 vs. 19.78, *P* = 0.07). Figure 2c,d shows RANK expression on primary tumor and adjacent normal tissue. Additional file 3 shows other examples of different staining intensities of RANK. Among pregnant patients, a slightly lower RANK expression was observed in patients diagnosed in the first trimester of pregnancy (Figure S2c,d in Additional file 2).

**Figure 3:**
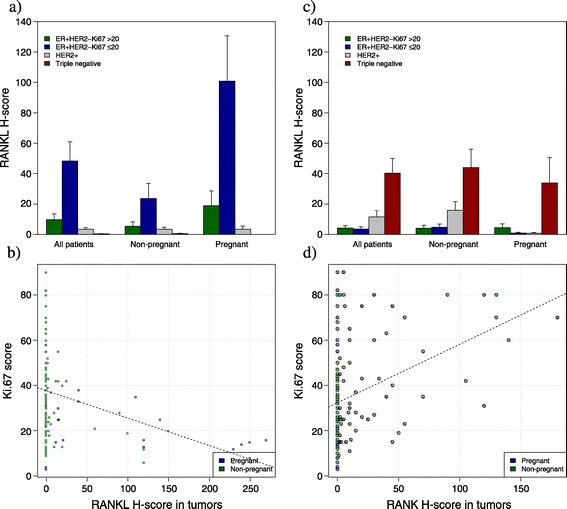
**Expression of RANK and RANKL according to breast cancer subtype and correlation with Ki67. (a)** Expression of RANKL by immunohistochemistry using the H-score (*y* axis) according to breast cancer subtypes in all patients, nonpregnant patients and pregnant patients. RANKL expression was higher in luminal A tumors (ER+, HER2–, Ki67 < 20%) compared with all other subtypes, particularly in pregnant patients (*P* <0.0001), than in nonpregnant patients (*P* = 0.32). **(b)** Negative correlation between Ki67 score by immunohistochemistry (*y* axis) and RANKL H-score (*x* axis) (*P* <0.0001, Pearson correlation = –0.29). **(c)** Expression of RANK by immunohistochemistry using the H-score (*y* axis) according to breast cancer subtypes in all patients, nonpregnant patients and pregnant patients. RANK expression was higher in triple-negative tumors compared with all other subtypes in all patients (*P* <0.0001), nonpregnant patients (*P* < 0.0001) and pregnant patients (*P* = 0.05). **(d)** Positive correlation between Ki67 score by immunohistochemistry (*y* axis) and RANK H-score (*x* axis) (*P* <0.0001, Pearson correlation = 0.37). ER, estrogen receptor; HER2, human epidermal growth factor receptor 2; RANK, receptor activator for nuclear factor κB; RANKL, receptor activator for nuclear factor κB ligand.

Table 1 shows the association between RANK and RANKL expression on the primary tumor and clinicopathological features. For pregnant and nonpregnant patients, RANKL expression was higher in small (*P* = 0.01), well-differentiated (*P* <0.001) and PgR-positive tumors (*P* <0.001). On the contrary, higher RANK expression was observed in patients with poorly differentiated and hormone receptor-negative tumors (all *P* <0.001). RANKL expression was significantly higher in luminal A-like tumors (mean H-score: 45.45) compared with other subtypes, with the lowest expression observed in triple-negative subtypes (mean H-score: 0.23) (Figure 3a). Furthermore, the RANKL H-score was negatively correlated with Ki67 (*P* < 0.001) (Figure 3b). On the other hand, RANK expression was significantly higher in triple-negative tumors (mean H-score: 40.91) with the lowest expression observed in luminal A tumors (mean H-score: 4.68) (Figure 3c). A positive correlation was observed between RANK H-score and Ki67 (Figure 3d).

To evaluate factors independently associated with RANKL and RANK expression, an adjusted linear regression model showed that only pregnancy and high PgR expression were independently associated with high tumor RANKL expression (both *P* <0.001). On the other hand, breast cancer subtypes and administering neoadjuvant chemotherapy were independently associated with RANK expression (*P* <0.001) (Table 2).

**Table 2:** **Linear regression model showing clinicopathological factors that are independently associated with RANK and RANKL expression**

	**Independent association with increasing RANKL expression (** ***P*** **value)**	**Independent association with increasing RANK expression (** ***P*** **value)**
Diagnosis during pregnancy (yes vs. no)	<0.001	0.67
Tumor size (≤2 cm vs. >2 cm)	0.23	0.87
Nodal involvement (negative vs. positive)	0.15	0.19
Histological grade (I vs. II vs. III)	0.09	0.7
Breast cancer subtypes (luminal A vs. luminal B vs. HER2 vs. triple negative)	0.75	<0.001
Increasing progesterone receptor expression by IHC (continuous variable)	<0.001	0.17
Neoadjuvant chemotherapy (yes vs. no)	0.69	<0.001

HER2, human epidermal growth factor receptor 2; IHC, immunohistochemistry; RANK, receptor activator for nuclear factor κB; RANKL, receptor activator for nuclear factor κB ligand.

### Genes and pathways associated with RANK and RANKL expression on primary breast tumors

**Figure 4:**
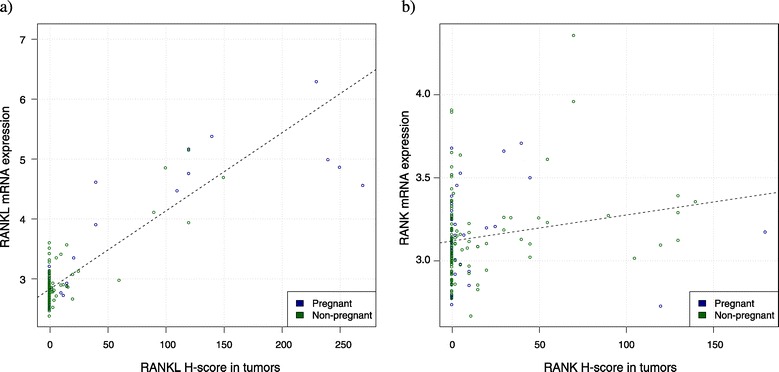
**Correlation between RANK and RANKL H-score and mRNA levels. (a)** Positive correlation between RANKL mRNA expression (*y* axis) and RANKL H-score by immunohistochemistry (*x* axis) (*P* <0.0001, Pearson correlation = 0.89). **(b)** Positive correlation between RANK mRNA expression (*y* axis) and RANK H-score by immunohistochemistry (*x* axis) (*P* = 0.01, Pearson correlation = 0.19). RANK, receptor activator for nuclear factor κB; RANKL, receptor activator for nuclear factor κB ligand.

Using false discovery rate <0.05, out of 18,665 evaluated genes 1,207 genes and 151 genes were significantly correlated (either positively or negatively) with RANK and RANKL H-score, respectively (Additional file 4). Almost perfect correlation was observed between tumor RANKL H-score and mRNA levels (*r* = 0.89, *P* < 0.001) (Figure 4a). A weaker correlation, albeit significant, was observed between tumor RANK H-score and mRNA levels (*r* = 0.19, *P* = 0.012) (Figure 4b). GSEA showed that tumors expressing high levels of RANKL by immunohistochemistry had activated pathways; of particular relevance were those related to bone resorption, mammary gland development, regulation of chemotaxis and T-cell proliferation. On the other hand, high RANK expression was associated with several activated pathways, of relevance to immune response, and proliferation-related pathways (Additional file 5). These results were consistent even after adjusting the analysis for pregnancy status.

### Association between RANK and RANKL expression on the primary tumor and disease-free survival

At a median follow-up of 65 months, 41.5% of the pregnant patients (*n* = 27) and 26.1% of the nonpregnant patients (*n* = 34) developed a disease-free survival event. Neither RANK nor RANKL were associated with the outcome when considering pregnant patients, nonpregnant patients or both groups combined (Additional file 6).

## Discussion

This is the first study to evaluate the expression of RANK and RANKL in young and pregnant breast cancer patients. We found that pregnancy significantly increases RANKL expression both on the tumor and adjacent normal epithelial tissue. We also found that RANKL expression is associated with activation of important cancer-related pathways.

Recently, Pfitzer and colleagues reported on RANK/RANKL expression in primary breast tumors analyzing 601 core biopsies collected from the neoadjuvant GeparTrio phase III trial, using the same antibodies and staining procedures as in the current analysis [14]. However, unlike our study where we did not use cutoff points to define RANK/RANKL positivity, Pfitzer and colleagues used an H-score cutoff value ≥8.5 to define patients with high expression. In their analysis, high RANK and RANKL expressions were observed in 14.5% and 6%, respectively. In the current study, RANK and RANKL H-scores ≥8.5 were observed in 28.5% and 12.3% respectively in the nonpregnant group and in 18.5% and 29.2% in the pregnant group. Previously, we reported higher RANKL mRNA expression in younger breast cancer patients [10]. The higher prevalence of RANK/RANKL expression in the current analysis could be due to the younger patient population in the current study (median age = 36 years) compared with the GeparTrio trial (median age = 49 years). It is important to note that in our analysis, RANK/RANKL expression was evaluated in surgical specimens as opposed to core biopsies (as in the GeparTrio trial). We have shown that both RANK and RANKL heterogeneously stain tumor and normal breast tissue (Additional files 1 and 3); therefore, it is also possible that the small core samples may underrepresent the incidence of RANK and RANKL expression.

We observed higher RANK expression in poorly differentiated and triple-negative tumors, with positive correlation between RANK H-score and Ki67 labeling index. Similar findings were also observed in the GeparTrio study and in earlier work using RANK mRNA expression [14,21]. This is consistent with preclinical work showing that RANK induces the expression of breast cancer stem and basal/stem cell markers [21]. On the other hand, we found higher expression of RANKL in the slowly proliferating luminal A-like tumors. Positive correlation was observed between RANKL H-score and PgR scores (Figure S5a in Additional file 7), which is consistent with the preclinical data showing co-expression of RANKL and PgR on the normal breast, pre-invasive lesions and invasive lesions [6].

The striking difference in RANKL expression between pregnant and nonpregnant patients is intriguing. RANKL expression was significantly higher in both the normal epithelial and neoplastic cells arising during pregnancy. These results are consistent with preclinical data showing high expression of RANKL in normal breast tissue during pregnancy [4]. However, this is the first report to evaluate RANKL expression in tumors diagnosed during pregnancy in the clinical setting. We also found that RANK and RANKL expression differ according to the pregnancy trimester (Additional file 2), which was also observed in preclinical experiments [4].

The gene expression analysis provided confirmatory data regarding the known functions of RANK and RANKL and insights into the potential role(s) of RANK and RANKL in breast carcinogenesis. We observed a positive correlation between immunohistochemistry and mRNA expression, confirming the robustness of the performed assays. We also determined significant correlation between the RANKL H-scores and genes known to be related to RANKL function such as *PgR* and parathyroid hormone-related protein (Figure S5b in Additional file 7). There was significant correlation between genes that we recently found to be related to breast cancer during pregnancy, such as insulin growth factor 1 and *TC1* (Figure S5c,d in Additional file 7), a positive regulator of the Wnt/beta-catenin signaling pathway [16]. Consistent with the known functions of RANKL, we observed activation of bone resorption and mammary gland development pathways in tumors with high RANKL expression. We also observed high expression of pathways related to T-cell proliferation. RANKL is known to enhance T-cell response and increase dendritic cell survival via binding to RANK [22]. RANKL expression on the tumor was associated with downregulation of proliferation and cell cycle-related pathways, which is consistent with the clinical correlations that we observed, in which high RANKL was mainly observed in the slowly proliferative tumors. On the other hand, RANK expression was associated with activation of immune response and proliferation, again consistent with the high expression of RANK in the poorly differentiated, triple-negative tumors in which immune-related pathways are emerging as important targets in this tumor subtype [23].

A limitation of our study is the small sample size, which might have masked a potential prognostic role of RANK or RANKL. Recently, neither was found to be predictive of pathological complete response in a large neoadjuvant study [14]. However, irrespective of the prognostic value of RANK and RANKL, their role as cancer targets is established. Another limitation is the lack of information on the phase of menstrual cycle at the time of tissue sampling, as evolving data suggest RANKL expression to vary across the menstrual cycle [7,24]. However, we believe that the striking differences in RANKL expression between pregnant and nonpregnant patients are unlikely to be significantly impacted by the unavailability of these data.

Our results underscore the relevance of RANKL as a potential target in breast cancer arising in young women. To further validate this concept, we are conducting a preoperative window trial evaluating the impact of the anti-RANKL denosumab on the biology of tumors arising in young women (D-BEYOND; NCT01864798). We also found that tumors diagnosed during pregnancy are more likely to express RANKL, raising the question of whether it could serve as a valid treatment target in these patients. To date we lack evidence supporting the use of denosumab in patients with primary breast cancer, yet several studies are currently ongoing to address this question including the predictive value of RANKL expression [25]. Another important finding is the high expression of RANKL in the normal breast of pregnant patients. Preclinical data support the important role of RANKL expression in breast cancer initiation, and given the known short-term risk of developing breast cancer following pregnancy it is plausible that RANKL expression on the normal breast would identify patients at high risk of developing breast cancer following pregnancy. This hypothesis is rather speculative and requires further investigation, but could potentially open a new venue for identifying high-risk women who are candidates for chemoprevention strategies.

## Conclusions

This is the first study to evaluate the expression of RANKL in young and pregnant breast cancer patients. Our results indicate that pregnancy significantly increases RANKL expression independent of PgR or other factors; both on the primary tumor and on normal breast tissue. High expression of RANKL is also associated with activation of important cancer-related pathways. Our findings confirm the preclinical evidence suggesting RANKL as a potential breast cancer treatment target; particularly in young women and pregnancy-associated tumors.

## Additional files

Additional file 1:
**Is Figure S1 showing representative immunohistochemistry (IHC) images illustrating the range of staining intensity and heterogeneity expression for RANKL.** The H-score method, as described in Methods, accounts for the heterogeneity in staining intensity and fraction of cells with any staining observed with RANKL IHC. Scores were recorded for the percent of cells that stained with intensity of 0, 1, 2, 3. An H-score was calculated as follows: (% cells of 1 intensity × 1) + (% cells of 2 intensity × 2) + (% cells of 3 intensity × 3) = H-score. The maximum H-score would be 100% of cells of intensity 3, which would be 300. The precise H-score calculation is included for each image of low-expressing, medium-expressing and high-expressing examples. The staining score for tumor cells and normal adjacent cells were recorded separately. Similar heterogeneity in RANKL staining intensity and fraction of positive cells was observed in both tumors and normal breast. (a) RANKL IHC of a breast tumor sample with relatively low expression (H-score = 21). (b). RANKL IHC of a breast tumor sample with medium expression (H-score = 60). (c). RANKL IHC of a breast tumor sample with high expression (H-score = 140). (b) and (c) represent the heterogeneous distribution of RANKL staining intensities within the same sample.
Additional file 2:
**Is Figure S2 showing (a) RANKL expression by immunohistochemistry on the primary tumor of pregnant patients according to trimester at breast cancer diagnosis.** *y* axis, mean H-score and 95% confidence interval. Tumor diagnosed in the third trimester had the lowest RANKL expression (trimester 1 + 2 vs. trimester 3, *P* = 0.04). (b) RANKL expression by immunohistochemistry on adjacent normal epithelial cells of pregnant patients according to trimester at breast cancer diagnosis. *y* axis, mean H-score and 95% confidence interval. RANKL expression was lowest in the third trimester (trimester 1 + 2 vs. trimester 3, *P* < 0.001). (c) RANK expression by immunohistochemistry on the primary tumor of pregnant patients according to trimester at breast cancer diagnosis. *y* axis, mean H-score and 95% confidence interval. Tumor diagnosed in the first trimester had the lowest RANK expression (trimester 1 vs. trimester 2 + 3, *P* = 0.4). (d) RANK expression by immunohistochemistry on adjacent normal epithelial cells of pregnant patients according to trimester at breast cancer diagnosis. *y* axis, mean H-score and 95% confidence interval. RANK expression was lowest in the first trimester (trimester 1 vs. trimester 2 + 3, *P* = 0.27).
Additional file 3:
**Is Figure S3 showing representative IHC images illustrating the range of staining intensity and heterogeneity expression for RANK.** IHC scoring was performed using the H-score method, which accounts for the heterogeneity in staining intensity and fraction of cells with any staining observed with both RANK and RANKL IHC. Scores were recorded for the percent of cells that stained with intensity of 0, 1, 2, 3. An H-score was calculated as follows: (% cells of 1 intensity × 1) + (% cells of 2 intensity × 2) + (% cells of 3 intensity × 3) = H-score. The maximum H-score would be 100% of cells of intensity 3 which would be 300. The precise H-score calculation is included for each image of low-expressing, medium-expressing and high-expressing examples. The staining score for tumor cells and normal adjacent cells was recorded separately. Similar heterogeneity in RANK staining intensity and fraction of positive cells was observed in both tumors and normal breast. (a) RANK IHC of a breast tumor sample with relatively low expression (H-score = 20). (b). RANK IHC of a breast tumor sample with high expression (H-score = 70). (c). RANK IHC of a breast tumor sample with very high expression (H-score = 140). This image represents RANK expression detection at three different staining intensities within the same sample.
Additional file 4:
**Is a table presenting genes associated with RANK and RANKL expression by immunohistochemistry using the H-score as a continuous variable.**

Additional file 5:
**Is Figure S4 showing gene-set enrichment analysis showing upregulated pathways associated with RANKL (a) and RANK (b) expression by immunohistochemistry using the H-score as a continuous variable.**

Additional file 6:
**Is a table presenting univariate Cox regression analysis evaluating the effect of RANKL and RANK expression on disease-free survival.**

Additional file 7:
**Is Figure S5 showing (a) positive correlation between** ***PgR*** **mRNA expression (** ***y*** **axis) and RANKL H-score by immunohistochemistry (** ***x*** **axis) (** ***P*** **<0.0001, Pearson correlation = 0.35).** (b) Positive correlation between parathyroid hormone-related hormone mRNA expression (*y* axis) and RANKL H-score by immunohistochemistry (*x* axis) (*P* <0.0001, Pearson correlation = 0.47). (c) Positive correlation between *IGF1* mRNA expression (*y* axis) and RANKL H-score by immunohistochemistry (*x* axis) (*P* <0.0001, Pearson correlation = 0.34). (d) Positive correlation between *TC1* mRNA expression (*y* axis) and RANKL H-score by immunohistochemistry (*x* axis) (*P* < 0.0001, Pearson correlation = 0.32).

## Abbreviations

ER: estrogen receptor
HER2: human epidermal growth factor receptor 2
IEO: European Institute of Oncology
PgR: progesterone receptor
RANK: receptor activator for nuclear factor κB
RANKL: receptor activator for nuclear factor κB ligand
