# Supplementary material for: RANK-ligand (RANKL) expression in young breast cancer patients and during pregnancy
Source: Breast Cancer Res. 2015 Feb 21;17:24. doi: 10.1186/s13058-015-0538-7 (PMC4374174; doi:10.1186/s13058-015-0538-7)

Supplemental Figure 1

a)

H-Score = 21

[(2% x Intensity 1) + (2% x Intensity 2) + (5% x Intensity 3)]

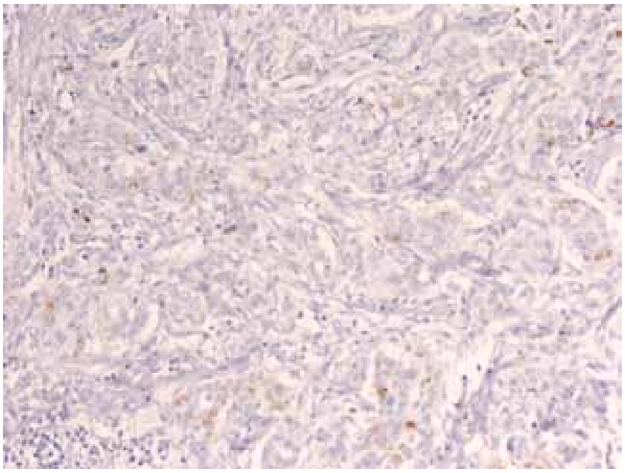

b)

H-Score = 60

[(10% x Intensity 1) + (10% x Intensity 2) + (10% x Intensity 3)]

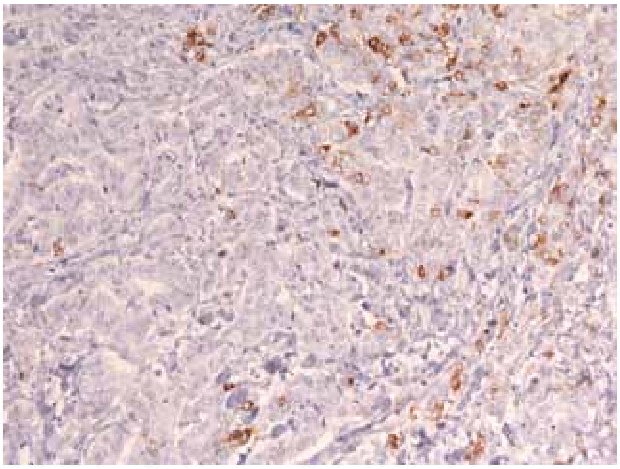

c)

H-Score = 140

[(20% x Intensity 1) + (20% x Intensity 2) + (30% x Intensity 3)]

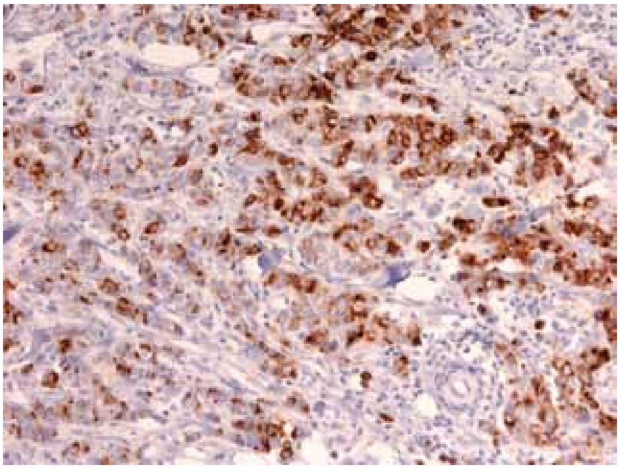

Supplement: Additional file 1: — Is Figure S1 showing representative immunohistochemistry (IHC) images illustrating the range of staining intensity and heterogeneity expression for RANKL. The H-score method, as described in Methods, accounts for the heterogeneity in staining intensity and fraction of cells with any staining observed with RANKL IHC. Scores were recorded for the percent of cells that stained with intensity of 0, 1, 2, 3. An H-score was calculated as follows: (% cells of 1 intensity × 1) + (% cells of 2 intensity × 2) + (% cells of 3 intensity × 3) = H-score. The maximum H-score would be 100% of cells of intensity 3, which would be 300. The precise H-score calculation is included for each image of low-expressing, medium-expressing and high-expressing examples. The staining score for tumor cells and normal adjacent cells were recorded separately. Similar heterogeneity in RANKL staining intensity and fraction of positive cells was observed in both tumors and normal breast. (a) RANKL IHC of a breast tumor sample with relatively low expression (H-score = 21). (b). RANKL IHC of a breast tumor sample with medium expression (H-score = 60). (c). RANKL IHC of a breast tumor sample with high expression (H-score = 140). (b) and (c) represent the heterogeneous distribution of RANKL staining intensities within the same sample. [file 13058_2015_538_MOESM1_ESM.pdf]
