# Supplementary figures and images for: RANK-ligand (RANKL) expression in young breast cancer patients and during pregnancy
Source: Breast Cancer Res. 2015 Feb 21;17:24. doi: 10.1186/s13058-015-0538-7 (PMC4374174; doi:10.1186/s13058-015-0538-7)

Supplemental Figure 2

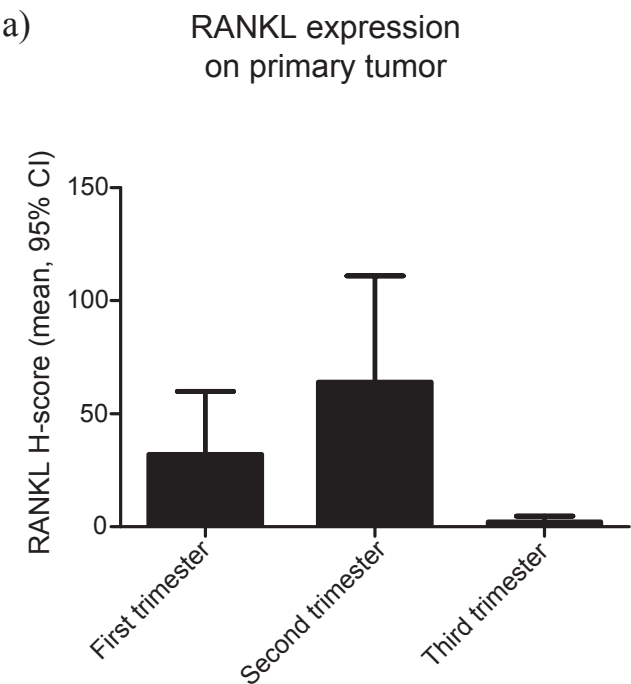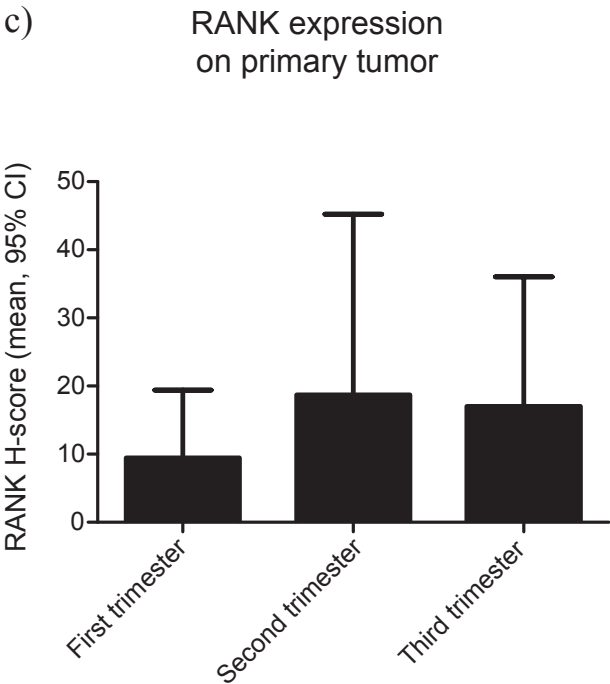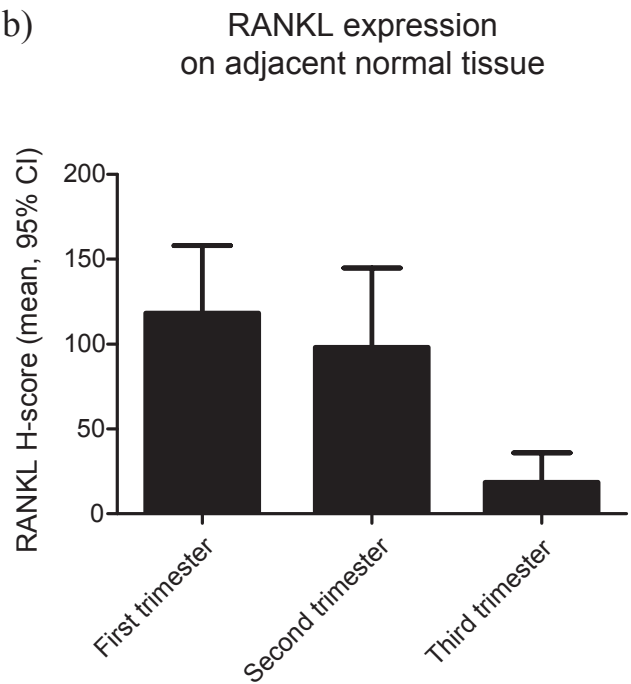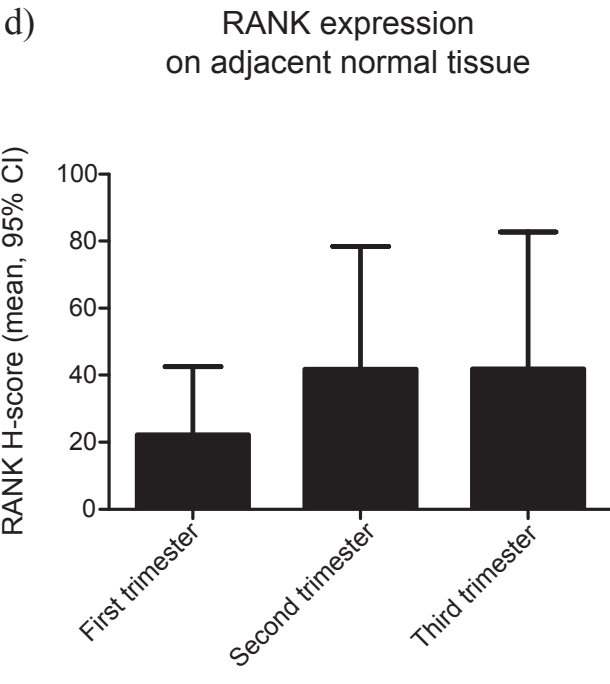

Supplement: Additional file 2: — Is Figure S2 showing (a) RANKL expression by immunohistochemistry on the primary tumor of pregnant patients according to trimester at breast cancer diagnosis. y axis, mean H-score and 95% confidence interval. Tumor diagnosed in the third trimester had the lowest RANKL expression (trimester 1 + 2 vs. trimester 3, P = 0.04). (b) RANKL expression by immunohistochemistry on adjacent normal epithelial cells of pregnant patients according to trimester at breast cancer diagnosis. y axis, mean H-score and 95% confidence interval. RANKL expression was lowest in the third trimester (trimester 1 + 2 vs. trimester 3, P < 0.001). (c) RANK expression by immunohistochemistry on the primary tumor of pregnant patients according to trimester at breast cancer diagnosis. y axis, mean H-score and 95% confidence interval. Tumor diagnosed in the first trimester had the lowest RANK expression (trimester 1 vs. trimester 2 + 3, P = 0.4). (d) RANK expression by immunohistochemistry on adjacent normal epithelial cells of pregnant patients according to trimester at breast cancer diagnosis. y axis, mean H-score and 95% confidence interval. RANK expression was lowest in the first trimester (trimester 1 vs. trimester 2 + 3, P = 0.27). [file 13058_2015_538_MOESM2_ESM.pdf]

Supplemental Figure 7

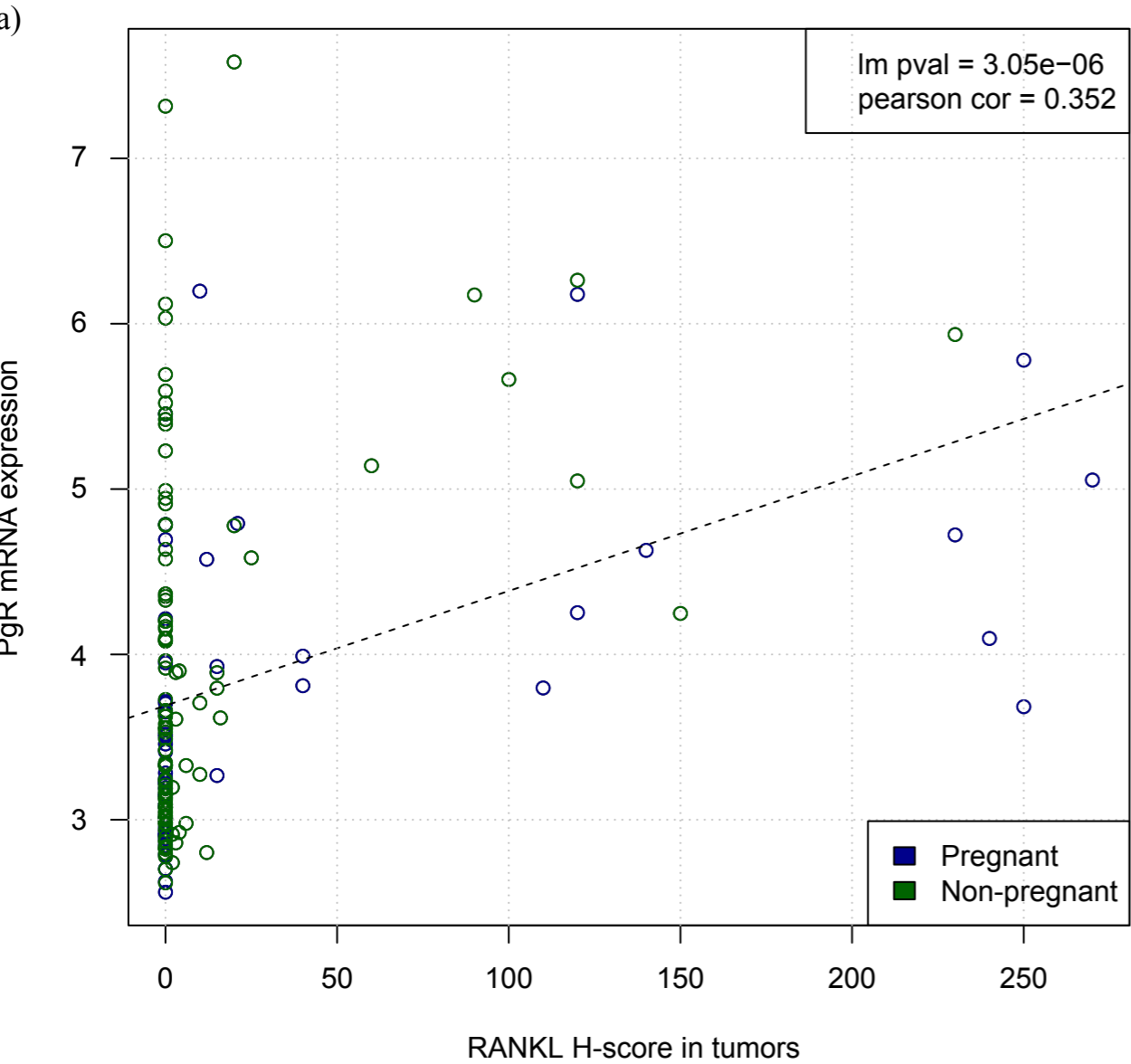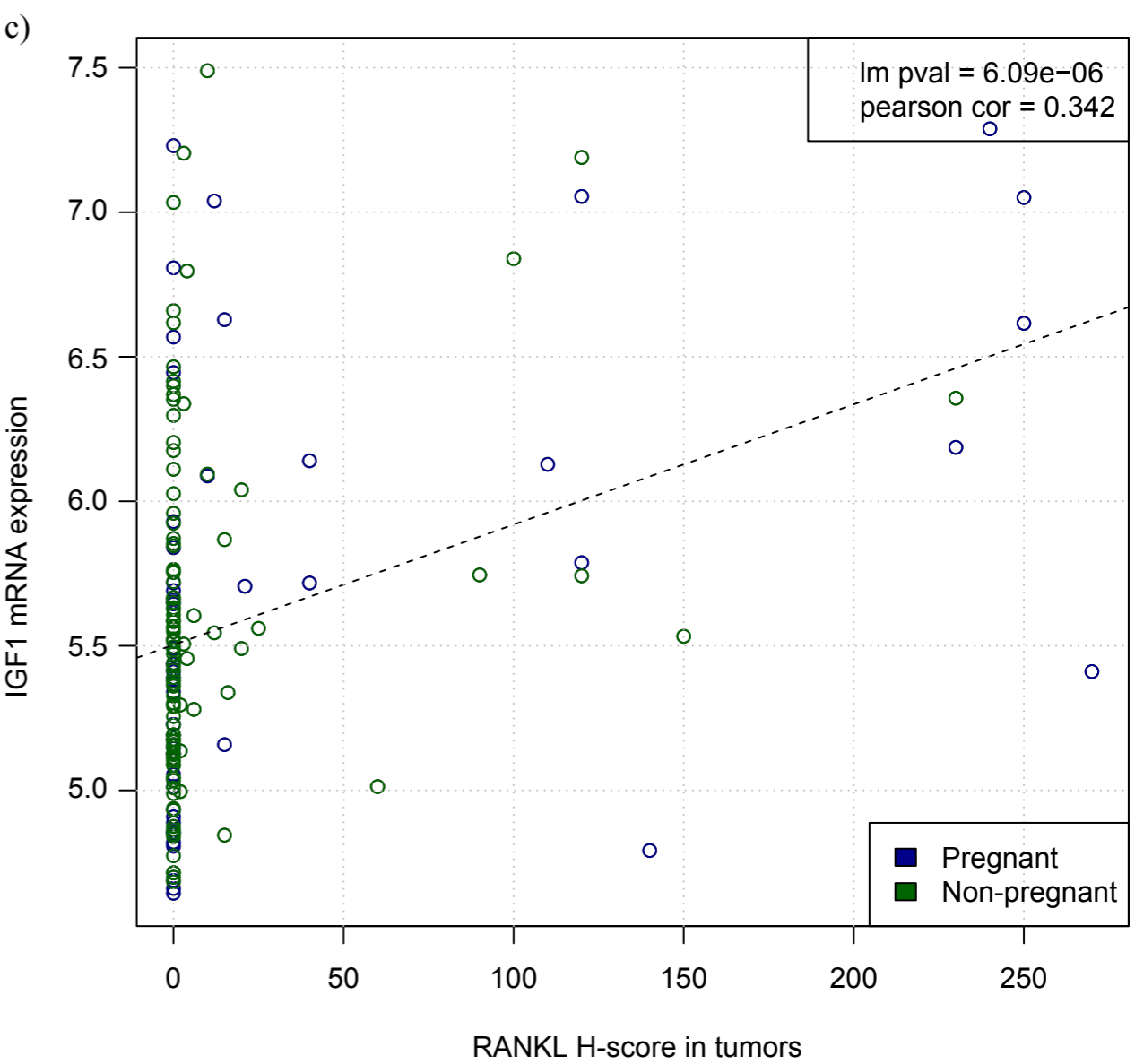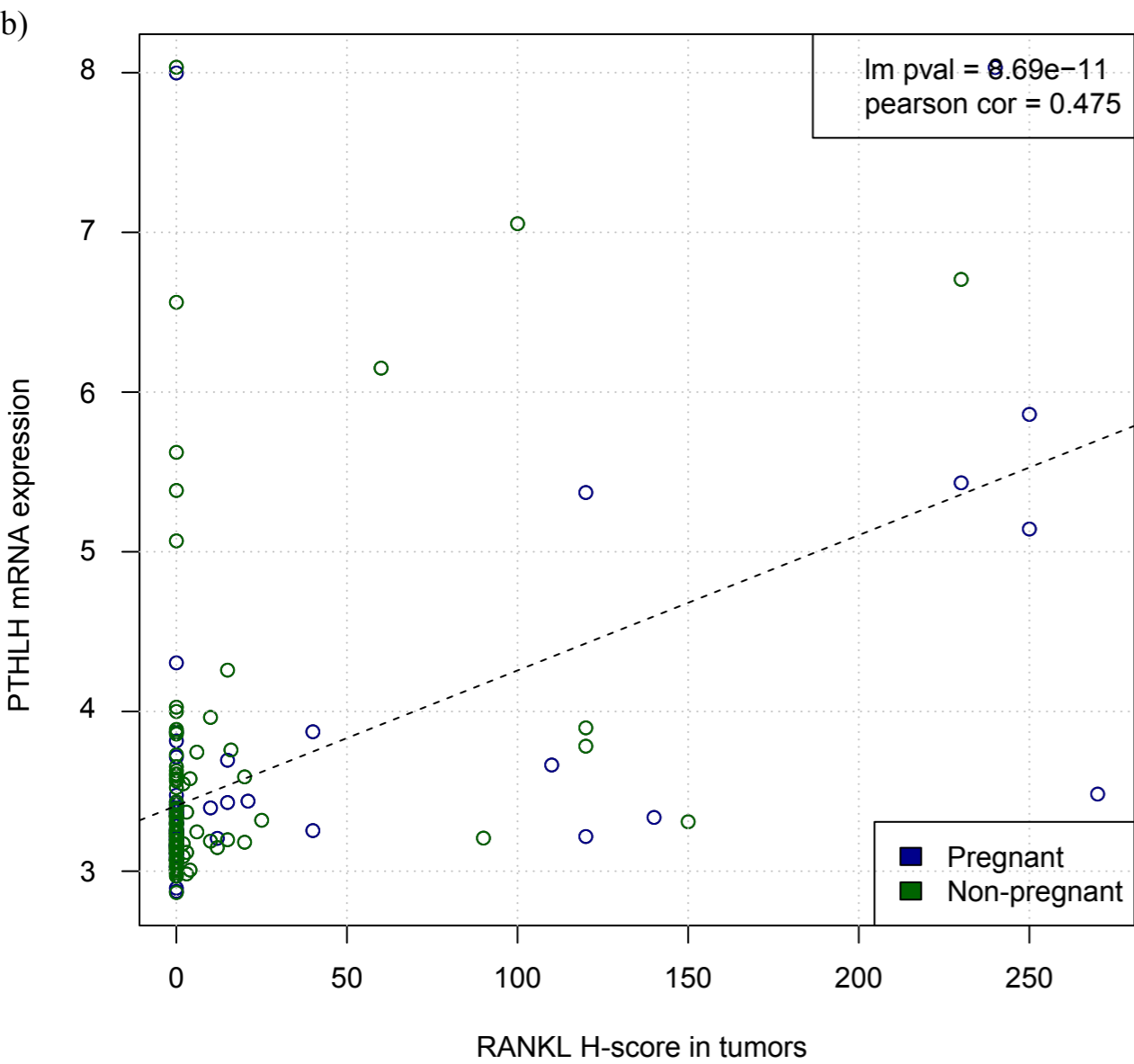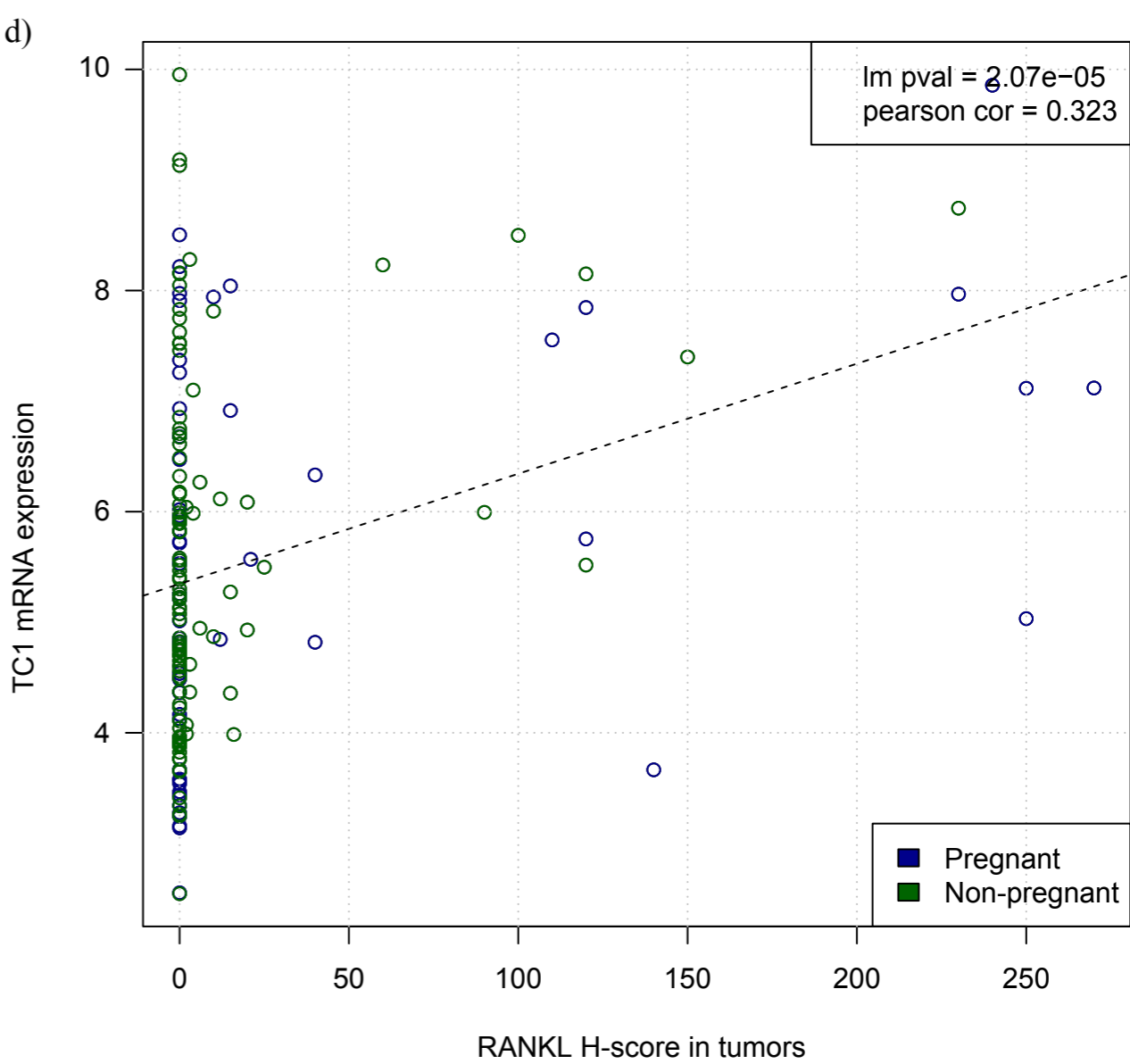

Supplement: Additional file 7: — Is Figure S5 showing (a) positive correlation between PgR mRNA expression ( y axis) and RANKL H-score by immunohistochemistry ( x axis) ( P <0.0001, Pearson correlation = 0.35). (b) Positive correlation between parathyroid hormone-related hormone mRNA expression (y axis) and RANKL H-score by immunohistochemistry (x axis) (P <0.0001, Pearson correlation = 0.47). (c) Positive correlation between IGF1 mRNA expression (y axis) and RANKL H-score by immunohistochemistry (x axis) (P <0.0001, Pearson correlation = 0.34). (d) Positive correlation between TC1 mRNA expression (y axis) and RANKL H-score by immunohistochemistry (x axis) (P < 0.0001, Pearson correlation = 0.32). [file 13058_2015_538_MOESM7_ESM.pdf]
