# Supplementary material for: RANK-ligand (RANKL) expression in young breast cancer patients and during pregnancy
Source: Breast Cancer Res. 2015 Feb 21;17:24. doi: 10.1186/s13058-015-0538-7 (PMC4374174; doi:10.1186/s13058-015-0538-7)

Supplemental Figure 3

a)

H-Score = 20

(20% x Intensity 1)

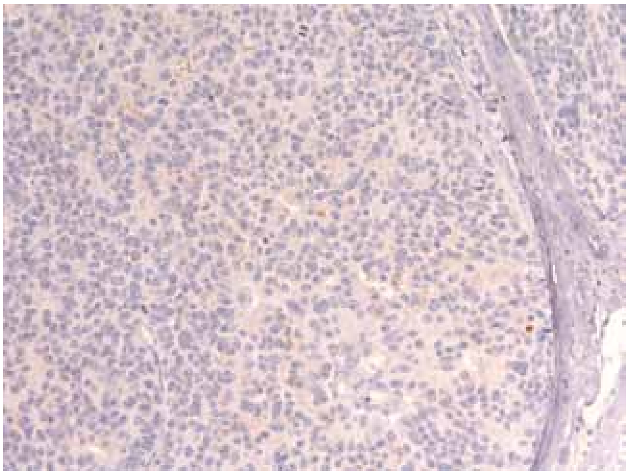

b)

H-Score = 70

[(30% x Intensity 1) + (20% x Intensity 2)]

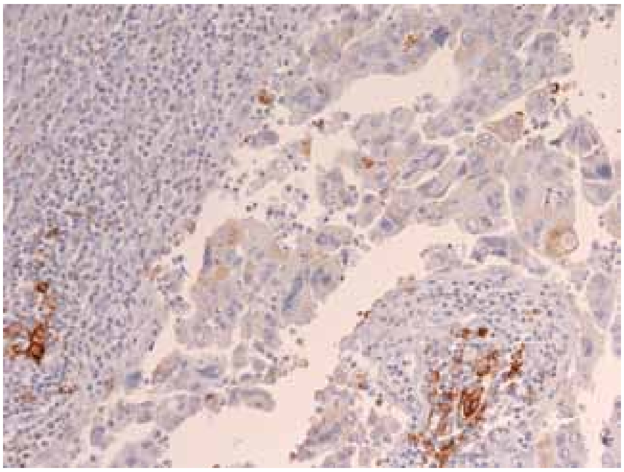

c)

H-Score = 140

[(40% x Intensity 1) + (20% x Intensity 2) + (20% x Intensity 3)]

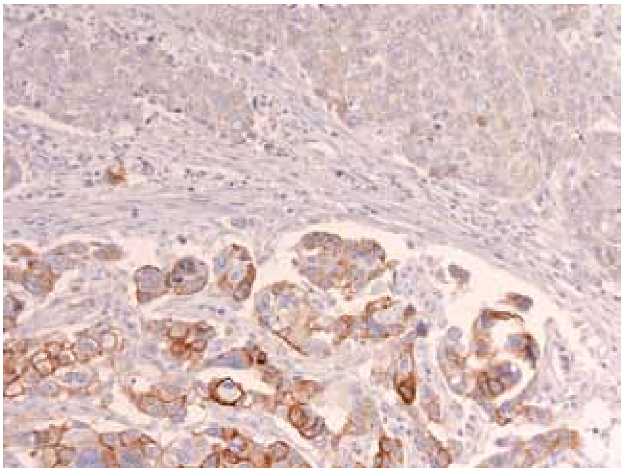

Supplement: Additional file 3: — Is Figure S3 showing representative IHC images illustrating the range of staining intensity and heterogeneity expression for RANK. IHC scoring was performed using the H-score method, which accounts for the heterogeneity in staining intensity and fraction of cells with any staining observed with both RANK and RANKL IHC. Scores were recorded for the percent of cells that stained with intensity of 0, 1, 2, 3. An H-score was calculated as follows: (% cells of 1 intensity × 1) + (% cells of 2 intensity × 2) + (% cells of 3 intensity × 3) = H-score. The maximum H-score would be 100% of cells of intensity 3 which would be 300. The precise H-score calculation is included for each image of low-expressing, medium-expressing and high-expressing examples. The staining score for tumor cells and normal adjacent cells was recorded separately. Similar heterogeneity in RANK staining intensity and fraction of positive cells was observed in both tumors and normal breast. (a) RANK IHC of a breast tumor sample with relatively low expression (H-score = 20). (b). RANK IHC of a breast tumor sample with high expression (H-score = 70). (c). RANK IHC of a breast tumor sample with very high expression (H-score = 140). This image represents RANK expression detection at three different staining intensities within the same sample. [file 13058_2015_538_MOESM3_ESM.pdf]
