# Supplementary material for: RANK-ligand (RANKL) expression in young breast cancer patients and during pregnancy
Source: Breast Cancer Res. 2015 Feb 21;17:24. doi: 10.1186/s13058-015-0538-7 (PMC4374174; doi:10.1186/s13058-015-0538-7)

Supplemental Figure 5

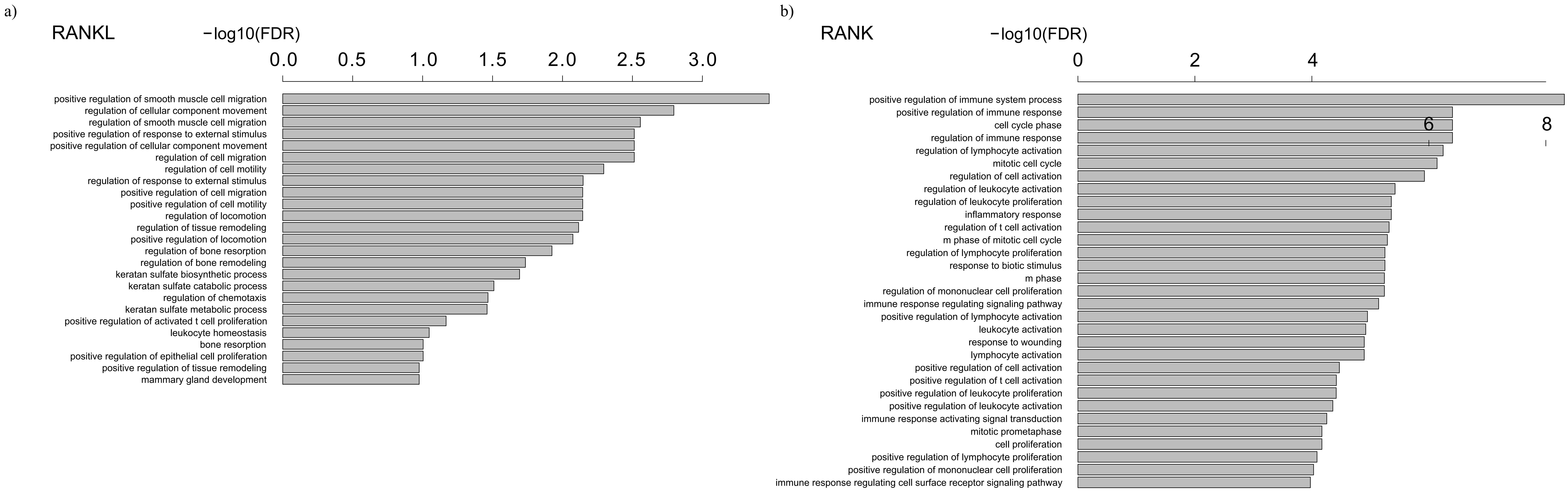

Supplement: Additional file 5: — Is Figure S4 showing gene-set enrichment analysis showing upregulated pathways associated with RANKL (a) and RANK (b) expression by immunohistochemistry using the H-score as a continuous variable. [file 13058_2015_538_MOESM5_ESM.pdf]
