# Supplementary material for: RANK-ligand (RANKL) expression in young breast cancer patients and during pregnancy
Source: Breast Cancer Res. 2015 Feb 21;17:24. doi: 10.1186/s13058-015-0538-7 (PMC4374174; doi:10.1186/s13058-015-0538-7)
Supplement: Additional file 6: — Is a table presenting univariate Cox regression analysis evaluating the effect of RANKL and RANK expression on disease-free survival. [file 13058_2015_538_MOESM6_ESM.docx]

**Supplemental file 6.**

|  |  | **HR (95% CI)** | ***P*-value** |
| --- | --- | --- | --- |
| **All patients** (N=195) | RANK | 0.97 (0.94 – 1.0) | 0.15 |
|  | RANKL | 0.97 (0.93 – 1.0) | 0.32 |
| **Pregnant patients** (N=65) | RANK | 0.95 (0.88 – 1.0) | 0.28 |
|  | RANKL | 0.99 (0.99 – 1.0) | 0.52 |
| **Non-pregnant patients** (N=130) | RANK | 0.97 (0.94 – 1.0) | 0.15 |
|  | RANKL | 0.97 (0.93 – 1.0) | 0.32 |
